# Potential confounding factors in currently used antibiotic susceptibility assays for the honey bee pathogen Melissococcus plutonius

**DOI:** 10.1099/jmm.0.002109

**Published:** 2025-12-12

**Authors:** Peter Fowler, Robyn Hawley, Meghan O. Milbrath

**Affiliations:** 1 Comparative Medicine and Integrative Biology, College of Veterinary Medicine, Michigan State University, East Lansing, MI, USA; 2 College of Veterinary Medicine, Michigan State University, East Lansing, MI, USA; 3 Department of Entomology, Michigan State University, East Lansing, MI, USA

**Keywords:** antibiotic resistance, European foulbrood, honey bee, oxytetracycline, USA

## Abstract

**Introduction.** *Melissococcus plutonius* is the causative agent of European foulbrood (EFB), a disease of honey bees that is endemic in many areas of the USA. Only one antibiotic, oxytetracycline (OTC), is approved for EFB management, and there have been reports of recalcitrance.

**Gap Statement.** Resistant strains of *M. plutonius* have been identified in Canada and Japan, but methodology differs between studies, making reliable comparisons difficult. Additionally, no *M. plutonius* isolates from the USA have yet been tested for susceptibility to OTC, despite decades of use.

**Aim.** Here, we determine the impact of media, time and persistence on the results of commonly used growth and antibiotic resistance assays using regionally representative *M. plutonius* isolates.

**Methodology.** Twelve genetically diverse isolates of *M. plutonius* were tested for susceptibility to OTC using previously published assays, but with variations in media and time to determine factors that may be impacting results.

**Results.** Media composition and incubation time dramatically impact antibiotic susceptibility assays for *M. plutonius*, differing widely between strains, likely due to differences in OTC stability. Assays that ended when growth appeared on antibiotic-free agar showed that all strains remained susceptible to OTC with an MIC of 2–4 µg ml^−1^. However, *M. plutonius* remains viable after OTC efficacy wanes, with some strains able to persist at room temperature for at least 3.5 years.

**Conclusion.** To standardize antibiotic susceptibility testing for *M. plutonius,* we recommend the use of M110 media due to stability and speed of growth. However, all strains of *M. plutonius* persist on M110 beyond the window of OTC efficacy, complicating assay results and interpretation, and additional research is needed to determine the clinical implications of these findings.

**Impact Statement**

Here, we present several important factors that will help refine and standardize antibiotic resistance screening for *Melissococcus plutonius*. We additionally show that no resistance is evident in the genetically diverse isolates we have tested, but all isolates are able to persist beyond the point of antibiotic efficacy, with some isolates persisting in the environment for many years. This has significant implications for both treatment recommendations and the study of transmission, which are critical for managing this disease.

## Data Summary

All supporting data can be found in the supplementary material. GenBank accession numbers for isolates used in this study: GCF_049530735.1, GCF_049530715.1, GCF_049530775.1, GCA_049530435.1, GCA_051042715.1, GCA_049530035.1, GCA_049531295.1, GCA_049529855.1, GCA_049529955.1, GCA_049531355.1, GCA_049531195.1 and GCA_049530575.1. GenBank accession numbers and strain identification for isolates are listed in Table S1.

## Introduction

The European honey bee (*Apis mellifera*) is important to US food production, with pollination services providing an estimated 17 billion dollars in increased crop yield annually [1]. However, honey bees are under increased stress from pesticide exposure, parasites and diseases, which together account for a loss of nearly half the colonies each year [2]. One of the most pervasive diseases affecting honey bees is European foulbrood (EFB), a bacterial disease affecting honey bee brood which has become endemic in some regions of the USA [3,4]. Previous cross-sectional surveillance carried out by our lab revealed very high rates of EFB, with 33% of colonies infected [4]. Colonies afflicted with this disease experience a significant reduction in colony growth [5], causing an unsustainable economic burden to beekeepers [6].

The causative agent of EFB is a unique, single-genus, single-species bacterium called *Melissococcus plutonius*. Recent genomic studies have revealed that variants of this bacterium fall into two broad categories: the first, called ‘typical’, known to be sensitive to oxygen and variable in virulence and the second, called ‘atypical’, known to be oxygen-tolerant and highly virulent to larvae *in vitro* [7,12]. Strains are further divided into three clonal complexes (CC) based on multi-locus sequence typing (MLST) [13], with CC3 and CC13 in the typical grouping and CC12 representing the atypical grouping; each clonal complex comprises many sequence types. Characterization of the full diversity of *M. plutonius* has been hampered by its fastidious and stochastic behaviour *in vitro*, with isolates varying dramatically in growth characteristics [14].

In the USA, the only antibiotic labelled for management of EFB is oxytetracycline (OTC), which has been in use since the 1950s [15,17]. Resistance to OTC has been well documented in the USA for another honey bee bacterial disease, American foulbrood [3,18] (caused by *Paenibacillus larvae*), but nothing is known about the OTC susceptibility of US variants of *M. plutonius*. Beekeepers have few options to manage EFB, making OTC resistance an increasing concern.

Previous studies examining *M. plutonius* resistance to OTC included variants from multiple countries and reported all variants to be susceptible with a MIC of 1–2 µg ml^−1^ [17,19, 20]. Recently, Masood *et al*. reported an isolate from Canada with an OTC MIC of 16 µg ml^−1^ using both agar dilution and a microbroth dilution assay [21]. Similar findings were subsequently reported in Japan [22]. However, methodology differed significantly between these studies, including media composition (and pH), and incubation time, both factors that impact the stability of OTC [23,24]. Most notably, the study in Canada and Japan utilized a recently popularized medium for the growth of *M. plutonius*, brain heart infusion supplemented with potassium phosphate and soluble starch (KSBHI), while previous studies used a medium developed in the 1950s, commonly referred to as M110 [25].

Here, we test if OTC resistance can be found in *M. plutonius* isolates circulating in the USA. Given recent reports of resistant *M. plutonius* in Canada [21] and Japan [22], as well as reports of recalcitrance to treatment [26], we hypothesized that resistant strains would be prevalent among US beekeeping operations. We additionally characterize the impact of methodological variation in hopes of developing a consistent and repeatable assay. Results show that media composition, time and corresponding OTC degradation have significant implications on outcomes. Additionally, we show no evidence of OTC resistance in isolates from the USA, with all isolates having an MIC below 4 µg ml^−1^; however, all isolates persist on media well beyond the efficacy of high concentration OTC, and we further demonstrate that some isolates can survive on some materials for multiple years. We provide recommendations for standardizing this assay that can be deployed at scale to ensure treatment and dosage are appropriate for managing this important disease.

## Methods

### Isolates used in this study

Three isolates of *M. plutonius* from four MLST sequence types were selected for testing, representative of the diversity among US beekeeping operations identified in previous studies [4,5, 27]. GenBank accession numbers and references for each isolate are listed in Table S1 (available in the online Supplementary Material). *M. plutonius* ATCC 35311 (type strain) was included in all assays to ensure repeatability. All isolates were field collected between 2019 and 2023 and stored in M110 broth containing 20% glycerol at −80 °C.

### Media composition and preparation of bacterial inoculum

Media used in this study, referred to as KSBHI and M110, were prepared as previously described [25] with slight modifications. Basal media is another medium commonly used in previous *M. plutonius* studies, but it is not commonly used in our lab and was not included in this analysis. For 1 L KSBHI, the following are added to 900 ml deionized water: 37 g brain heart infusion (Research Products International Corp. (RPI), Mount Prospect, IL, USA), 10 g soluble starch and *15 g KH_2_PO_4_ [MilliporeSigma (Sigma-Aldrich), Burlington, MA, USA] and stirred under heat to dissolve. Deionized water is then added to a total volume of 1 L. The pH is then adjusted to *6.7 using a 5M solution of KOH. For agar, 15 g of agar is added prior to autoclaving. Asterisk marks the differences from previously published KSBHI media [25].

For 1 L M110, the following are added to 960 ml deionized water: 2.5 g peptone [Oxoid Ltd. (Oxoid), Basingstoke, Hampshire, UK], *1 g d-glucose (RPI), 2 g soluble starch (Becton, Dickinson and Company (BD), 7 Loveton Circle, Sparks, MD, USA), 2.5 g yeast extract (Oxoid), 5 g neopeptone (BD), 2 g trypticase peptone (Oxoid), 50 ml 1M phosphate buffer (pH 6.7) stirred under heat to fully dissolve. The pH is similarly adjusted to *6.7 using 5M KOH. For agar, 15 g agar (RPI) is added prior to autoclaving. After autoclaving, 0.4 g l-cysteine HCl (Sigma-Aldrich) is dissolved in 1 ml deionized water and filter sterilized into the media using a 22-µm PVDF syringe filter (Sigma-Aldrich). Asterisk marks the differences from previously published M110 media [25].

Bacterial inoculum was prepared according to recommendations for anaerobic bacteria outlined by Clinical and Laboratory Standards Institute (CLSI) M11 [28], harvesting fresh colonies to prevent selecting for certain phenotypes reported in previous *M. plutonius* studies [29]. Briefly, each isolate was streaked onto freshly prepared M110 agar plates and incubated under anaerobic conditions (85% N_2_, 10% CO_2_, 5% H_2_) at 37 °C using the Thermo-Forma anaerobic system Model 1025 [Thermo Fisher Scientific (Forma), Waltham, MA, USA] and checked daily for growth. After growth was observed, a single colony visually consistent with *M. plutonius* and less than 24 h old was spread across a freshly prepared M110 agar plate and grown as previously described, checking daily for growth. Again, when colonies matured and were less than 24 h old, a sterile swab was used to harvest colonies from the agar surface into sterile PBS without calcium or magnesium (DPBS) (Sigma-Aldrich) to a turbidity of 0.5 McFarland Units (McF) read by DEN-1B densitometer, ~1–3×10^6^ c.f.u. ml^−1^ as confirmed by culture of serial dilutions. CLSI recommendations use McF. To show a linear relationship of McF to optical density (OD at 600 nm) used in other studies, ten dilutions of typical and atypical *M. plutonius* were made in triplicate, and McF and OD were measured for each, with six McF having an approximate OD of 0.5, while one McF had an OD of around 0.1 (Fig. S1).

### Growth curves

To generate growth curves for each isolate, 2 ml of fresh broth media (M110 and KSBHI) in round-bottom glass tubes was inoculated with 2 µl of bacterial suspension prepared as described above and incubated anaerobically at 37 °C. Initial turbidity readings in McF were taken using a DEN-1B densitometer at time zero and repeated every 6 h until growth had stabilized. Each reading was preceded by vortexing, and readings were taken after the culture had stabilized. Inoculum-free broth negatives and *M. plutonius* ATCC 35311 (type strain) were included as controls (Fig. S2). Readings from bacteria-free controls for each medium were subtracted from the turbidity readings to account for the impact of media components on density.

### Agar dilution assay for oxytetracycline

M110 and KSBHI agar plates were prepared with twofold dilutions of OTC from 128 to 2 µg/ml and inoculated by spotting 2 µl fresh culture suspensions described above. The position of spots on the plates was randomized to help control for the impact on growth. Plates were photographed every 24 h, and photographs were read by two independent blinded observers using CLSI guidelines to determine MIC, defined as the lowest concentration where growth was inhibited. Light haze or several individual colonies were not considered growth. The mean MIC at each timepoint was determined by averaging the two readings. All assays were repeated in triplicate, with at least 24 h separating each assay. Viability and bacterial identity were confirmed by subculturing a colony from the corresponding agar dilution plate into 5 ml freshly prepared KSBHI and culturing under anaerobic conditions at 37 °C for 7 days. Culture was then spread onto an M110 plate in four quadrants and incubated for 7 days. A single colony was then selected with a sterile toothpick and directly subjected to duplex PCR as described by Arai *et al.*, confirming typical and atypical strains, respectively [30].

Agar dilution assays were repeated with KSBHI and M110 prepared at multiple time points prior to inoculation to determine if media can be prepared in bulk for high-throughput assays. This included media prepared at 10 and 15 days prior to inoculation. To determine the impact of temperature on antibiotic efficacy, a set of dilution plates was prepared and stored in the incubation chamber at 37 °C under anaerobic conditions for 11 days prior to inoculation. The media prepared 10 and 15 days prior to inoculation were kept at 4 °C until use. The remaining agar dilution assay was performed as previously described, on both M110 and KSBHI, including negatives and the typical *M. plutonius* control strain ATCC 35311 (type strain).

### Assessment of environmental persistence

Bacterial persistence was assessed for atypical *M. plutonius* isolate 20F (ST19) and typical isolate ATCC 35311 (type strain) on wood and cloth to gain insights into how the bacteria may persist on hive material and bee suits, respectively. Briefly, surfaces were sterilized by autoclaving, inoculated with 20 µl *M*. *plutonius* in KSBHI at a concentration of 1.21×10^7^ c.f.u. ml^−1^ as determined by plate count, allowed to dry and placed in a sterile Whirl-Pak (Nasco Sampling LLC, a Filtration Group Company, Pleasant Prairie, WI, USA) open to air. At various time points, 100 µl sterile DPBS was added to the Whirl-Pak and agitated to dislodge bacteria and c.f.u. ml^-1^ was enumerated using a standard culture-based serial dilution assay. To assess survival within diseased larval remains, larval smears were prepared, as described by Hornitzky and Wilson[31], from diseased hives sampled in 2021 and placed in a cardboard slide box at room temperature for 3.5 years. To cultivate from larval smears, ~100 µl sterile DPBS was used to rehydrate the smear, and 1 µl was streaked onto freshly prepared M110 agar and incubated as previously described.

### Bioinformatics

Isolates previously underwent DNA extraction as described in Fowler *et al.* [4] and whole-genome sequencing on Illumina NovaSeq with 150 bp paired-end reads. The Bacterial and Viral Bioinformatics Resource Center (BV-BRC) was used for all bioinformatic analysis [32]. Reads were assembled *de novo* using the BV-BRC Genome Assembly service. This includes read trimming with Trim Galore! [33], followed by read assembly using Unicycler [34] as a SPAdes [35] optimizer. Draft assemblies were compared using the BV-BRC Bacterial Genome Tree service, including reference assemblies from other studies deposited on National Center for Biotechnology Information (NCBI). This analysis includes PGFams [32] for homology groups and the use of RAxML [36,37] to align nucleotide and amino acid sequences from single-copy genes. Codon trees were exported to iTOL [38] for formatting and annotated using Adobe Illustrator v. 24.1.2.

### Statistical analysis

All analysis was carried out in R v. 4.3.1 [39]. Figures were generated using ggplot2 [40]. To determine the impact of time on agar dilution assay results, only data for M110 were used, as degradation of KSBHI may confound results (Fig. S3). All MIC readings were pooled for each timepoint: 48 h, 72 h and 96 h, starting with MIC of 2 µg ml^−1^ OTC, as this represents growth on agar containing no antibiotic. Non-parametric Kruskal–Wallis tests were used for all group comparisons, followed by Dunn’s test for post hoc comparisons, as assumptions of normality could not be satisfied. Differences with a *P*-value of less than 0.05 were considered statistically significant. The half-life of OTC in media was determined using linear regression analysis of MIC data. OTC half-life analysis was restricted to bacterial isolates that demonstrated complete growth inhibition at high concentrations (MIC ≥256 µg ml^−1^) to avoid conflation between MIC and minimum bactericidal concentration in more resistant strains. MIC values were determined using twofold serial dilutions ranging from 2 to 128 µg ml^−1^, with values of 0 indicating no growth at 2 µg ml^−1^ and values of 256 indicating growth at 128 µg ml^−1^. Data were log-transformed prior to analysis to account for the exponential decay of antibiotic activity. While the Breusch–Pagan test indicated homoscedastic variance (BP=0.39, *P*=0.53), some deviation from normality was observed in the residuals (Shapiro–Wilk test, *W*=0.87, *P*<0.001), which was expected given the discrete nature of the twofold dilution series used in MIC determination.

## Results

We characterized the impact of media composition (KSBHI and M110) on the growth dynamics of 12 isolates of *M. plutonius* grouped by MLST sequence type, evaluating the time until growth on both M110 and KSBHI to determine the minimum time needed to evaluate antibiotic susceptibility assays. We used these values to determine the MIC of our isolates to OTC and subsequently the half-life of this antibiotic in agar media.

**Fig. 1.**
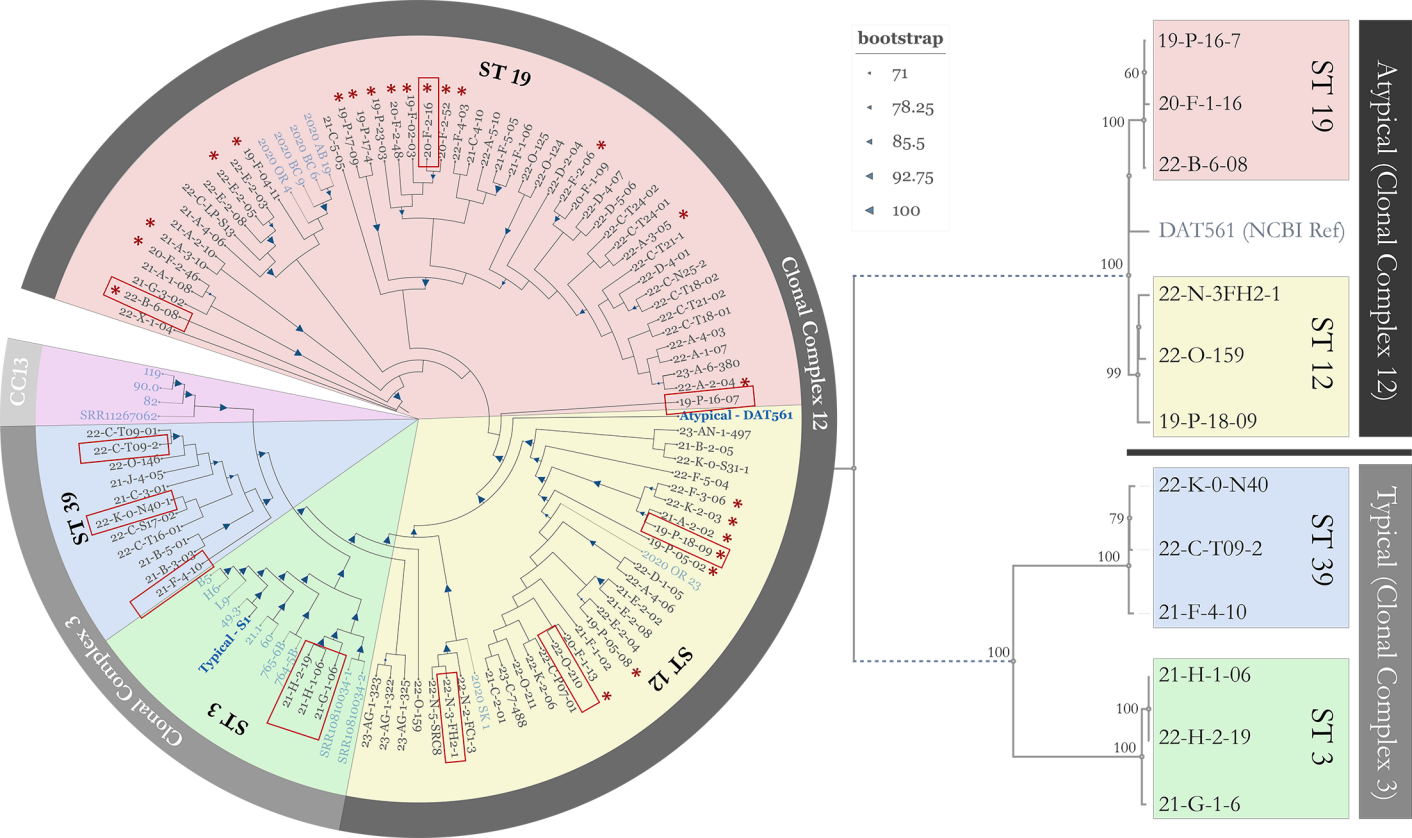
Core genome phylogeny of 91 *M*. *plutonius* isolates from US apiaries collected between 2019 and 2023. Isolates with blue text published from previous studies were retrieved from NCBI. Isolates used in this study are outlined in red, and phylogeny between study isolates is outlined on the right. DAT561 and S1 represent atypical and typical reference strains, respectively, obtained from NCBI. Numbers and arrows represent bootstrap values.

Core genome MLST analysis of 91 isolates collected by our lab from 26 beekeepers and multiple states between 2019 and 2023 revealed 4 sequence types as classified by MLST: ST3 and ST39 (clonal complex 3, typical) and ST12 and ST19 (clonal complex 12, atypical) [13]. To represent the diversity within our dataset, three isolates from different clades were chosen from each sequence type (Fig. 1). In addition to these 12 isolates (Table S1), *M. plutonius* type strain ATCC 35311 (ST1, clonal complex 13, typical) was also included in all assays.

### Media composition and media age significantly impact growth dynamics (M110 compared to KSBHI broth and agar)

**Fig. 2.**
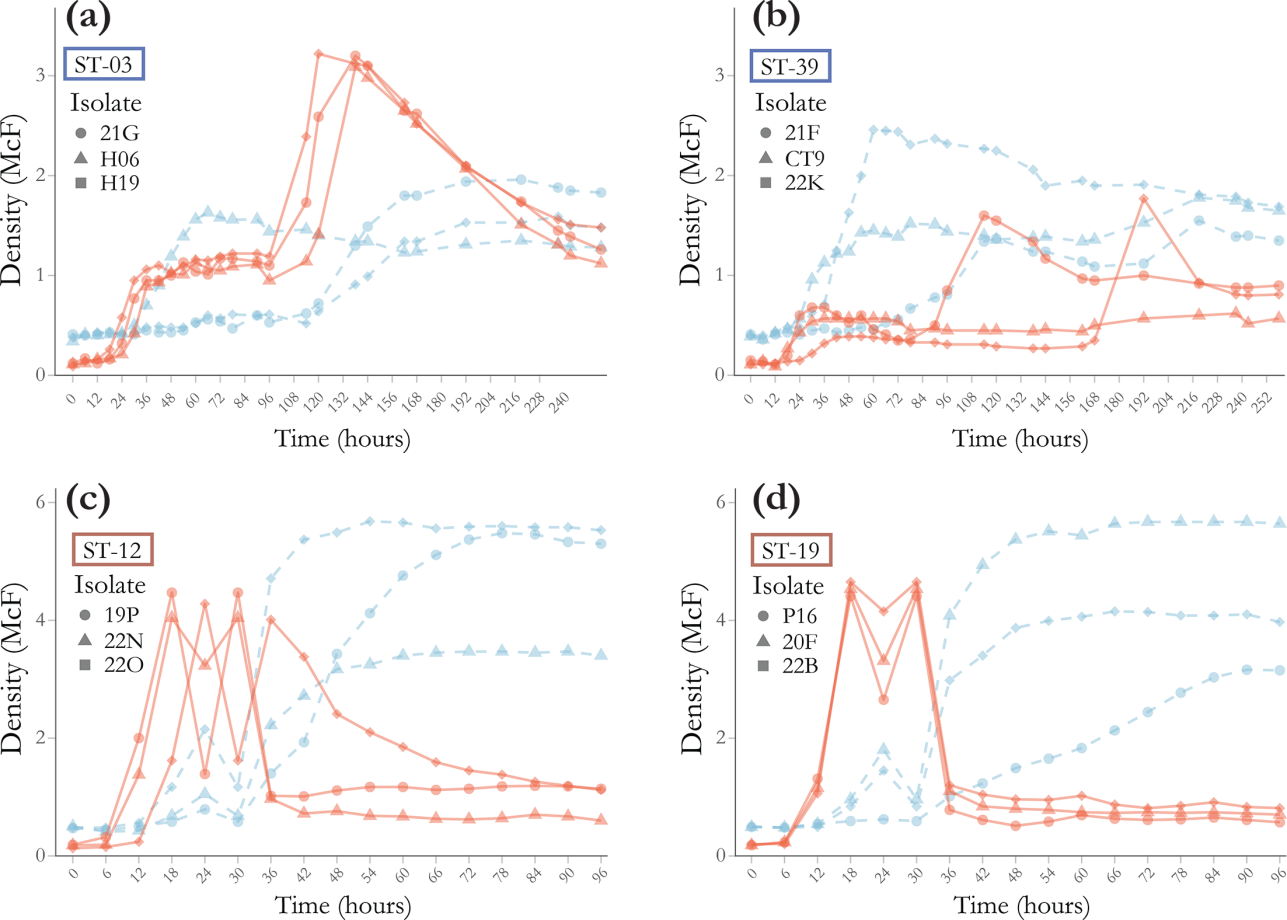
Growth of 12 *M*. *plutonius* sequence types used in this study on M110 broth (red) and KSBHI broth (blue), with density (McF) read every 6 h. Shapes correspond to the specific isolate ID listed. (**a**) typical ST3, (**b**) typical ST39, (**c**) atypical ST12 and (**d**) atypical ST19.

Growth in broth media for typical strains varied dramatically between both sequence type and media type (KSBHI vs. M110). In M110 broth, a bi-phasic growth curve is evident in both ST-03 (Fig. 2a) and ST-39 (Fig. 2b), with turbidity increasing until around 36 h post-inoculation, before reaching a stationary phase. For ST-03, this phase lasted ~30 h for all three isolates but varied within isolates in ST-39, with one isolate remaining in the stationary phase for the entire 258 h and another being in the stationary phase for 102 h. Following this stationary phase, turbidity increased significantly before declining again to approximately the same turbidity as reached in the initial stationary phase. In the KSBHI broth, typical strains also showed more erratic growth within isolates. Two of the isolates in ST-03 had a lag phase of 108 h before growth, while the other had a lag phase of only 24 h. Sequence type 39 showed more rapid growth early on in KSBHI broth for two strains, but the third showed growth characteristics similar to the same strains grown in M110 broth (Fig. 2b). The typical type strain, ATCC 35311, produced normal growth curves in both broth media (Fig. S2) with a stable stationary phase.

Atypical strains (ST12 and ST19) had the most consistent growth patterns between the three selected isolates (Fig. 2c and d) but growth differed drastically between M110 broth and KSBHI broth. When grown in M110 broth, all isolates reached early log phase at 12 h except for one ST-12 isolate, which had slightly deferred initial growth. Turbidity peaked at 18 h post-inoculation. Turbidity for all atypical isolates decreased at the following reading, followed by another increase 6 h later and a sharp decline 6 h after that to a near-zero turbidity. This pattern was nearly identical between ST-19 isolates but differed slightly for the ST-12 isolate 22O (Fig. 2d). In KSBHI broth, growth appeared to consist of an initial spike at 24 h post-inoculation, followed by a decline at 30 h. Turbidity then increased logarithmically for all isolates except 20F (Fig. 2d), which had a slow, near-linear growth pattern. Atypical strains all showed a similar pattern in KSBHI broth, interestingly showing an early peak at 24 h, followed by a decline before entering log phase growth at 36 h, which mostly stabilized by hour 60 of the study.

*M. plutonius* growth on agar provided more consistent results than growth in broth for both media types. All isolates from ST3, ST12 and ST19 showed growth at 48 h on M110 agar. Isolates within ST39 differed in the time until growth was established on M110 agar, with isolate CT9 growing by 48 h, but some replicates of 21F and 22K took 72–96 h before growth appeared. All isolates were able to grow on KSBHI agar by 72 h; however, KSBHI agar prepared in advance shows a reduced capacity to support the growth of *M. plutonius*. KSBHI plates stored at 37 °C for 11 days had significantly impaired growth in all sequence types (Fig. S3), while M110 agar shows no decline in viability, even after many months.

### Impact of incubation time and OTC half-life on M110 and KSBHI agar dilution assays

**Fig. 3.**
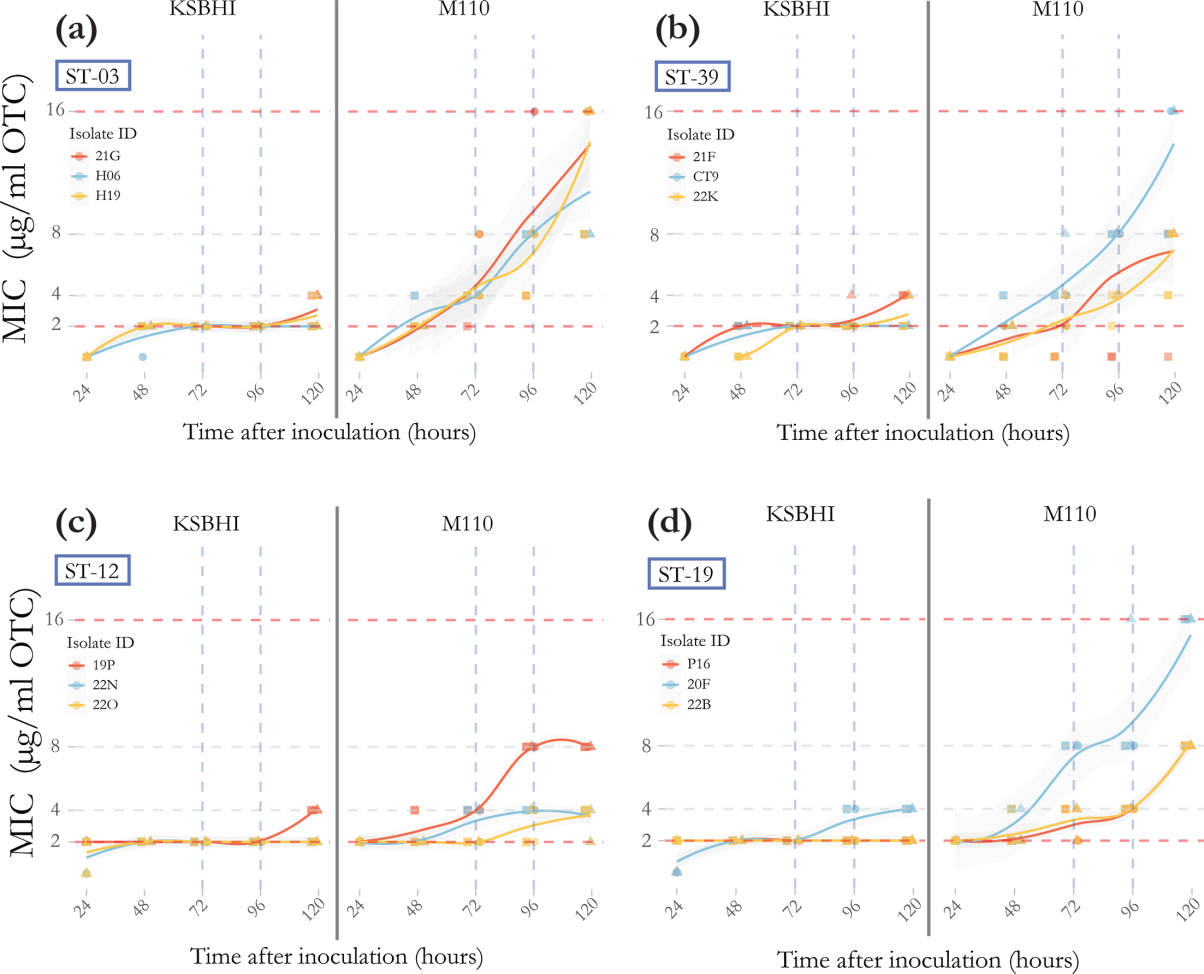
MIC of OTC (µg ml^−1^) for agar dilution assay read every 24 h using two different media, M110 (left) and KSBHI (right). Shapes represent the three replicates for each reading, and colour represents different isolates within each sequence type. Red dashed lines at MIC 2 represent when growth is first observed on the control plate containing no OTC, but not at 2 µg ml^−1^ OTC, while the dashed red line at MIC 16 represents when an isolate is considered resistant. Vertical dashed lines represent the two timepoints that this assay has been interpreted in previous studies [21,22]. (**a**) typical ST3, (**b**) typical ST39, (**c**) typical ST12 and (**d**) atypical ST19.

MIC of OTC for isolates growing on M110 agar changed significantly between 48, 72 and 96 h, suggesting that OTC efficacy begins to wane after 48 h at 37 °C (Fig. 3a) (*X*^2^ (2) = 22.254, *P*=1.47e−5). Trends of increasing MIC over time were similar across sequence types, with some variation in M110 agar for isolate CT9 (ST39) (Fig. 3b) and 20F (ST19) (Fig. 3d). Efficacy of OTC continues to wane at two and 3 weeks post-inoculation as *M. plutonius* begins to grow on plates with higher initial concentrations of antibiotic (Fig. S4). This loss of OTC efficacy can be observed on M110 agar, but not on KSBHI agar as, even if viable *M. plutonius* are present, the ability of KSBHI agar to support growth may be lost (Fig. S2). Interestingly, sequence type 12 may actually lose viability as none of the replicates at week 2 and 3 seem to increase MIC as seen in other sequence types on M110 agar (Fig. S4c). Using the MIC values for isolates that eventually grew on the highest concentration of OTC, the half-life of OTC in M110 agar media under incubation conditions (anaerobic at 37 °C) was determined to be 78.44 h using a generalized linear model on log_2_-transformed MIC values (*β*=0.0088, SE=0.00072, *P*<5.28×10⁻¹⁴). Plates stored at refrigeration temperature show similar growth dynamics to freshly prepared plates with little difference after 15 days (Fig. S5).

### Environmental persistence case study

Bacterial persistence was assessed for atypical *M. plutonius* isolate 20F (ST19) and typical type strain ATCC 35311 on wood and cloth to gain insights into how the bacteria may persist on hive material and bee suits, respectively. The longest that typical *M. plutonius* ATCC 35311 persisted on wood and cloth was 2 weeks, but none were recovered after 6 months at room temperature. Atypical isolate 20F (ST19) was shown to be viable on cloth and wood after 6 months at room temperature and after 2.5 years at 4 °C, with an estimated 1–4% of the original inoculum recovered. We also recovered an atypical isolate (ST12) from larval smears stored at room temperature after more than 3.5 years.

## Discussion

### Growth dynamics of typical and atypical *M. plutonius* varied widely depending on media composition

*M. plutonius* growth dynamics varied widely depending on media composition and MLST sequence type, with some showing variation between strains of the same sequence type, consistent with previous reports [14]. A 2020 study measured growth curves for 17 typical strains (7 from CC13 and 10 from CC3) using media with a similar composition to M110 (1% yeast extract, 1% glucose, 1% sucrose, 0.04% l-cysteine and 0.1 M KH_2_PO_4_, adjusted to pH 6.7). They also showed significant differences in growth between strains within the same MLST sequence type, and typical strains showed similar growth kinetics to typical strains in this study. Another recent study showed more consistent results within clonal complexes, but similar variability between clonal complexes [41]. Both these previous studies stopped measuring growth after 96 h. Here, we show a secondary growth occurring after 96 h for typical ST3 strains, which may suggest a transition in metabolism during later stages of growth in M110. Similar secondary growth was observed for two of the ST39 isolates, but there were marked differences within this sequence type, likely reflecting higher genetic diversity in ST39 when compared to ST3, which are nearly clonal (Fig. 1). Previous comparative genomics carried out by our group [4] showed that ST39 may represent a new clonal complex CC39, which is consistent with the growth differences from ST3 shown here. We only had one typical isolate representing CC13 (ATCC 35311) and growth curves in M110 and KSBHI are consistent with those reported by Kitamura *et al*. for CC13, showing normal lag, log and stationary phases in both media but growing to a much higher turbidity in M110 or basal when compared with KSBHI [41].

All atypical isolates grown in M110 showed a rapid increase in turbidity, followed by a temporary decrease before rebounding to the initial peak turbidity and finally losing nearly all turbidity, while growth in KSBHI produced a stable stationary phase. Auto-aggregation is occasionally observed in some strains, but this is seen more frequently in KSBHI and is unlikely to explain the rapid drop in turbidity seen in M110. Gram stains prepared from 24 h growth of ST12 (Fig. 2c) revealed increasing amounts of Gram-negative cells as turbidity decreased, consistent with compromised cell wall integrity (Fig. S6). Similar characteristics of atypical strains were also described by a recent study carried out using isolates from Japan [41]. They grew four different atypical sequence types in basal media [25], which is similar to M110 and also contains supplementary cysteine. They showed a similar loss of turbidity following initial growth. They additionally confirmed this reflects an actual drop in the concentration of viable cells through culture-based enumeration. When removing cysteine from this medium, they saw slower initial growth but a more subtle reduction in turbidity and hypothesized that cysteine may be related to observed cell lysis. However, increases in cysteine did not impact cell death, and it remains unclear why it induces rapid growth prior to cell lysis and what triggers this lysis. For atypical strains, reading assays prior to this lysis is critical to avoid conflating cell lysis due to media conditions with susceptibility to OTC. They also did not observe the drastic dip and rebound in turbidity shown here for atypical strains in M110. This could be due to genetic differences in *M. plutonius* used in each study. One of the atypical strains used in Japan, ST12 (DAT561), was included in the core genome phylogeny in Fig. 1, showing this strain to be distantly related to ST12 isolates used in this study. Given the brevity of the decrease in turbidity, it is also possible that this dip was missed, as readings were taken every 8 h instead of every 6.

The dramatic variation in growth shown here may have significant implications for previous studies of isolates, as media composition may be selecting for certain genetic variants in mixed infections. Additional research is needed to fully characterize the metabolic needs of *M. plutonius* variants, which may help explain observed deviations from expected growth phases.

### Incubation time and the impact on oxytetracycline efficacy

Here, we report a half-life for OTC in M110 media at a pH of 6.7 kept at 37 °C to be ~78 h. While OTC has been shown to remain stable in agar at low temperatures (Fig. S5) [42], degradation occurs rapidly at biological temperatures. According to previous research, the half-life is also heavily dependent on pH, with OTC in buffer at pH 7 showing a half-life of 26 h [43]. These results are consistent with the half-life reported in larvae in treated colonies [44] and likely reflect the biologically relevant concentrations during a standard course of treatment. However, this rapid decay of OTC presents a problem for standardizing an assay, especially with the erratic growth rates of *M. plutonius* in broth we report here. Agar produced more consistent rates of growth, but some isolates still take 72 h before growth is evident, when our agar plates can be expected to contain approximately half the initial concentration of OTC. This means that studies that report results at 72 h post-inoculation [22] can’t be compared to ones that report results at 96 h post-inoculation [21]. Given the bacteriostatic nature of OTC and the rapid decay under incubation conditions, the incubation time should be minimized, reading assays when growth on antibiotic-free media is first observed to avoid conflating antibiotic degradation with resistance. When we apply this parameter, no *M. plutonius* isolates in this study show any evidence of resistance to OTC, with all isolates having an MIC of 2–4 µg ml^−1^ or less. It should be noted that concentrations of OTC in media are approximations, based on preparation following CLSI guidelines, and may have been affected by variations in media preparation, such as the temperature of molten agar when OTC is added. In future research, the addition of internal quality control, such as the inclusion of a reference strain with an established MIC to OTC, will help ensure the accuracy and reliability of the results.

### The impact of media composition on oxytetracycline efficacy and assay results

In addition to incubation time, media composition significantly impacts the results of antibiotic susceptibility testing, which may be the result of OTC degradation. Interestingly, media composition has not been adequately considered in recent literature on *M. plutonius* antibiotic susceptibility [21,22] but was mentioned specifically in earlier studies [19,20] with regard to pH. The half-life of OTC is heavily dependent on pH, with more alkaline environments known to increase the rate of degradation [23]. The larval midgut pH is reported to be between 4 and 7 [45], but M110 media used to grow *M. plutonius* is commonly prepared at a pH of 7.2 [25]. KSBHI does not include any pH adjustment as part of standard protocols, so pH is unknown in previous studies. In this study, we adjusted the pH of both media to a slightly acidic and more biologically relevant pH of 6.7 and often found that prior to adjustment, the pH of KSBHI was closer to 5.9, a level that would be expected to dramatically increase the stability of OTC [43]. KSBHI is a less defined media when compared with M110, making comparisons between studies using KSBHI more difficult. M110 agar adjusted to a pH of 6.7 is shown here to be sufficient to grow all strains of *M. plutonius* rapidly and likely improves OTC stability over alkaline media or less defined media. Maintaining consistent media composition is important when interpreting these assays, as metallic cations and iron chelators, which are known to neutralize tetracyclines [24,46], are likely at different levels. We also noted lower MIC over longer periods on KSBHI (Fig. S4). The ability of KSBHI to grow some strains of *M. plutonius* was lost when stored anaerobically for 11 days at 37 °C. It is unclear if the lower MIC over time in KSBHI is due to increased OTC stability in this medium or the diminishing viability of KSBHI agar. Additional experiments storing KSBHI agar for shorter periods may help elucidate the role this plays in interpreting this assay.

### Persistence of *M. plutonius* and clinical implications

All isolates of *M. plutonius* were shown to survive on media containing concentrations of OTC far exceeding that encountered in treated honey bee colonies [44], which may explain beekeepers’ difficulty keeping this disease under control [26]. Antibiotic resistance is commonly referred to as a genetically inheritable trait that confers the ability to grow in the presence of high concentrations of an antibiotic [47]. Among the isolates studied here, we found no basis for resistance, but persistence or tolerance may play an important role in the ability of this pathogen to escape the effects of antibiotic applications. OTC is typically applied to honey bee colonies in powdered sugar or syrup, fed to adult bees for three consecutive treatments at 4–5-day intervals. This would give a potential window of application of around 15 days. Here, we demonstrate that all strains exposed to high levels of OTC remain viable after 15 days and will begin to grow *in vitro* (Fig. S4); however, additional research is needed to determine implications for treatment efficacy *in vivo*, where other factors, such as the microbiome, may impact results.

The ability of *M. plutonius* to remain viable in the environment has long been reported. In 1960, Bailey reported that *M. plutonius* remained viable in larvae smeared on a glass slide for at least 2 years [48]. Some strains are also known to persist for long periods in adult bees [49]. Here, we additionally report that atypical *M. plutonius* sequence type 19 grown in broth and allowed to dry on a surface remains viable for at least 3.5 years, which is surprising for a non-spore-forming bacterium [50,51] and holds significant implications for disease management. Using OTC in combination with other tools, such as shook swarm, may help increase efficacy and reduce re-emergence of disease in affected apiaries by removing this potential environmental reservoir. Additional research is needed to understand the mechanisms underlying this persistence and its relevance to clinical disease.

## Supplementary material

10.1099/jmm.0.002109 Uncited Fig. S1.

## Abbreviations

BV-BRC: Bacterial and Viral Bioinformatics Resource Center
CC: clonal complexes
CLSI: Clinical and Laboratory Standards Institute
EFB: European foulbrood
KSBHI: brain heart infusion supplemented with potassium phosphate and soluble starch
MLST: multi-locus sequence typing
NCBI: National Center for Biotechnology Information
OTC: oxytetracycline
