# Supplementary material for: Potential confounding factors in currently used antibiotic susceptibility assays for the honey bee pathogen Melissococcus plutonius
Source: J Med Microbiol. 2025 Dec 12;74(12):002109. doi: 10.1099/jmm.0.002109 (PMC12700387; doi:10.1099/jmm.0.002109)
Supplement: Uncited Fig. S1. [file jmm-74-02109-s001.pdf]

Supplementary Material

Table S1. Isolate data and NCBI WGS Accession Number. Three letter code is used throughout to simplify analysis and discussion. Raw Illumina reads are available at Bioproject ID: PRJNA1195964.

MLST Sequence Type	Isolate ID	3-Letter Code	GenBank Accession Number
ST 03	22-H-2-19	H19	GCF_049530735.1
	21-H-1-06	H06	GCF_049530715.1
	21-G-1-06	G21	GCF_049530775.1
ST 39	22-C-T09-2	CT9	GCA_049530435.1
	21-F-4-10	21F	GCA_051042715.1
	22-K-0-N40-1	22K	GCA_049530035.1
ST12	19-P-18-09	19P*	GCA_049531295.1
	22O-159	22O	GCA_049529855.1
	22-N-3-FH2-1	22N	GCA_049529955.1
ST19	19-P-16-07	P16*	GCA_049531355.1
	20-F-1-16	20F+	GCA_049531195.1
	22-B-6-08	22B	GCA_049530575.1

* Asterisk marks isolates collected in 2019 (Milbrath et al. 2021) [1]. + Plus marks an isolate collected in 2020 (Fowler et al. 2023) [2]. The remaining were isolated as part of cross-sectional surveillance between 2021-2023 (Fowler et al. 2025) [3].

References:

1. **Milbrath MO, Fowler PD, Abban SK, Lopez D, Evans JD**. Validation of Diagnostic Methods for European Foulbrood on Commercial Honey Bee Colonies in the United States. *Journal of Insect Science* 2021;21:6. DOI: 10.1093/jisesa/ieab075.

2. **Fowler PD, Schroeder DC, Kevill JL, Milbrath MOG**. No impact of hygienic behavior and viral coinfection on the development of European foulbrood in honey bee (*Apis mellifera*) colonies during blueberry pollination in Michigan. *Journal of Insect Science* 2023;23:21. DOI: 10.1093/jisesa/iead094.

3. **Fowler PD, Dhakal U, Chang JH, Milbrath MO**. Everything, everywhere, all at once - Surveillance and molecular epidemiology reveal Melissococcus plutonius is endemic among Michigan, US beekeeping operations of all sizes and present in some honey bee colonies year-round. 2025;2025.05.22.655465. DOI: 10.1101/2025.05.22.655465.

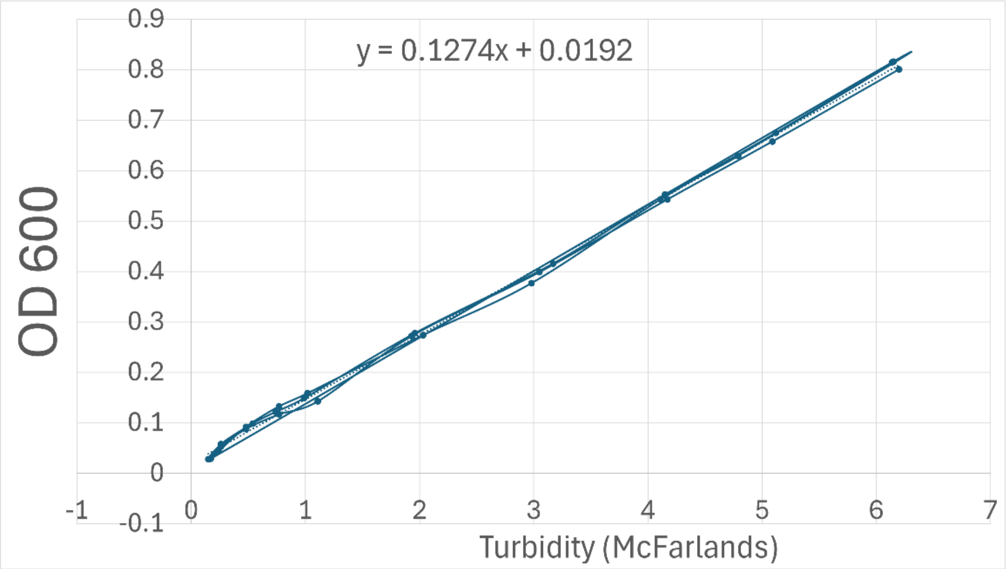

Figure S1. Optical density (600 nm) (OD) and turbidity (McF) of *M. plutonius* isolates suspended in sterile DPBS under differing dilutions. Data available in supplementary data file.

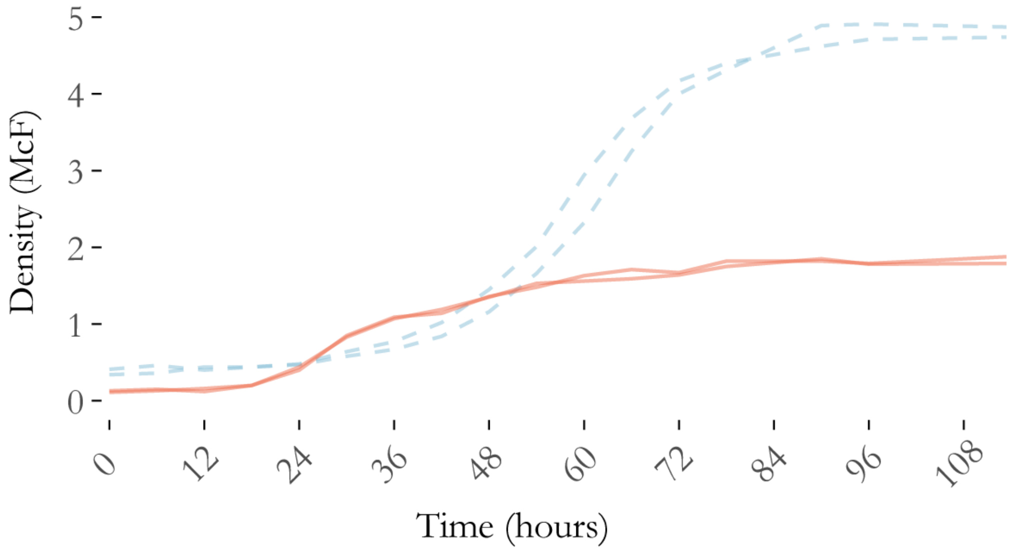

Figure S2. Growth of *M. plutonius* control strain ATCC 35311 in both KSBHI (blue dashed lines) and M110 (solid red lines) broth. Optical density was recorded in McF every 6 hours. Two lines represent two separate replicates.

Media age analysis (Figure S3)

It has been noted by our previous experience that KSBHI media loses its ability to grow *M. plutonius* unless freshly prepared, while M110 remains viable after preparation for at least a month, contrary to the literature [18]. To demonstrate this, KSBHI and M110 agar plates were prepared and placed in the anaerobic incubator at 37°C for 11 days. After 11 days, fresh plates of M110 and KSBHI were prepared and both sets were inoculated with a subset of two isolates from each sequence type as labeled in Figure B1. Inoculum preparation differed slightly from that outlined in the Methods section. Previously preserved isolates were streaked onto M110 agar and incubated at 37°C for under anaerobic conditions (10% CO_2, 5% H_2, 85% N_2) until colonies appeared. A single colony was suspended in freshly prepared KSBHI broth and incubated anaerobically until turbidity exceeded 0.1 OD. Broth was diluted in fresh KSBHI broth to a density of 0.05 OD and 2ul was used as inoculum. Plates were incubated anaerobically as stated above for 5 days and photographed (Figure S3). Three replicates were performed on separate occasions and each replicate included broth only negative, and ATCC 35311. Each replicate had three separate spots for each isolate.

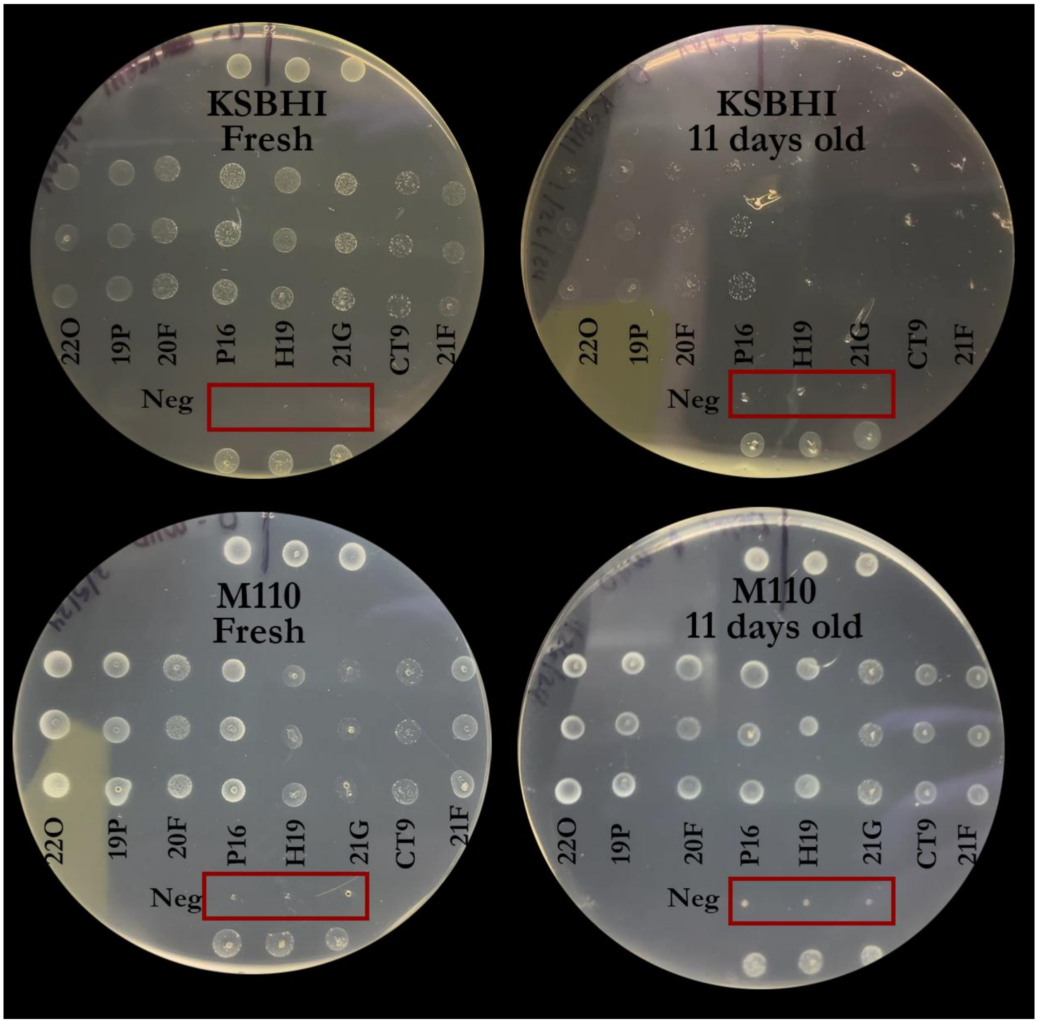

Figure S3. Growth of *M. plutonius* isolates on fresh and old media shows that KSBHI loses its ability to support growth, while M110 remains viable. Broth only negatives are outlined. Identical inoculums in triplicate are labeled. Three inoculations at the top of each plate are *M. plutonius* control strain ATCC 35311. This experiment was repeated on three separate occasions with similar results but photographs of replicate three are shown here as they had the most detail.

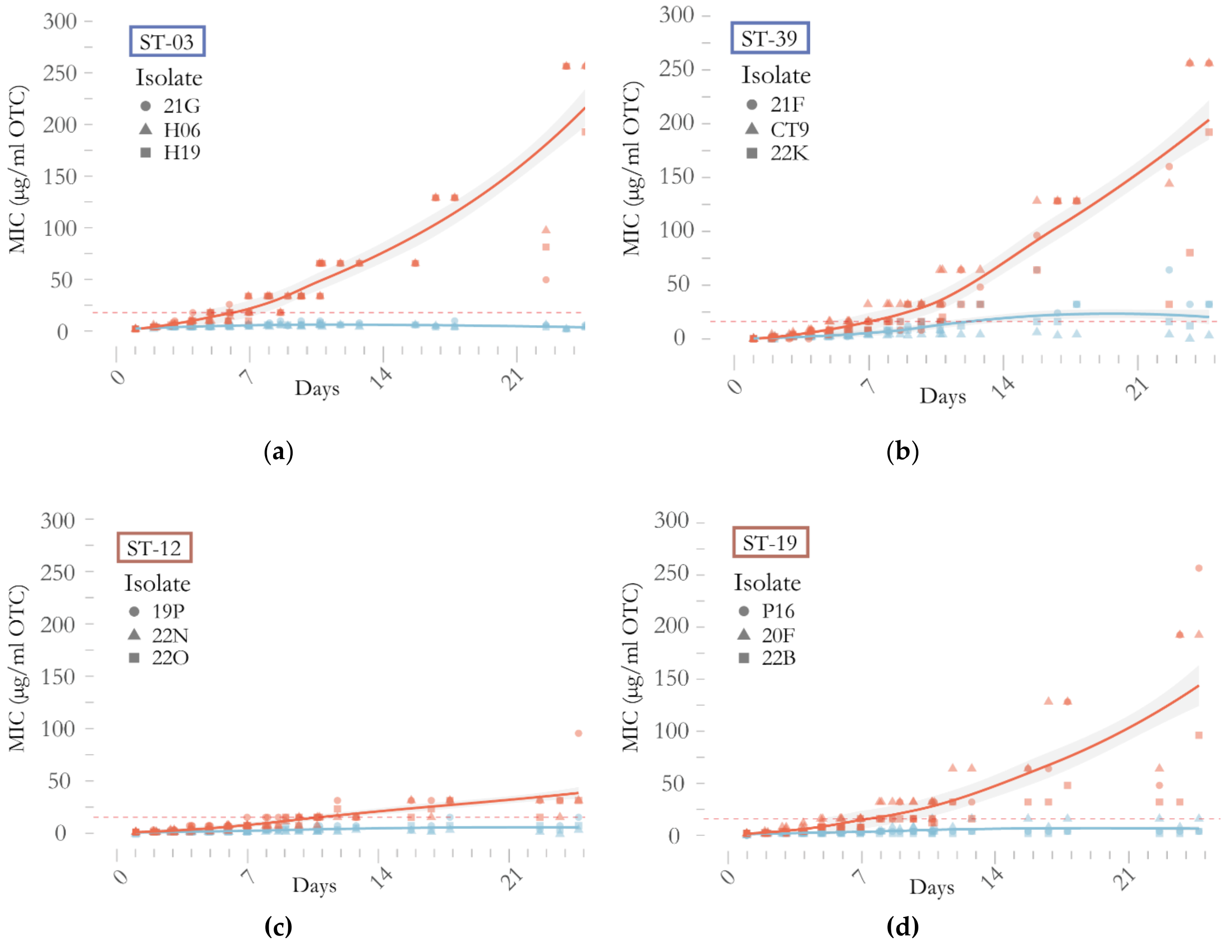

Figure S4. Minimum inhibitory concentration of oxytetracycline (µg/ml) for *M. plutonius* read every 24 hours on two different agar media, KSBHI (blue) and M110 (red). Each dot represents a triplicate with different shapes for each isolate.

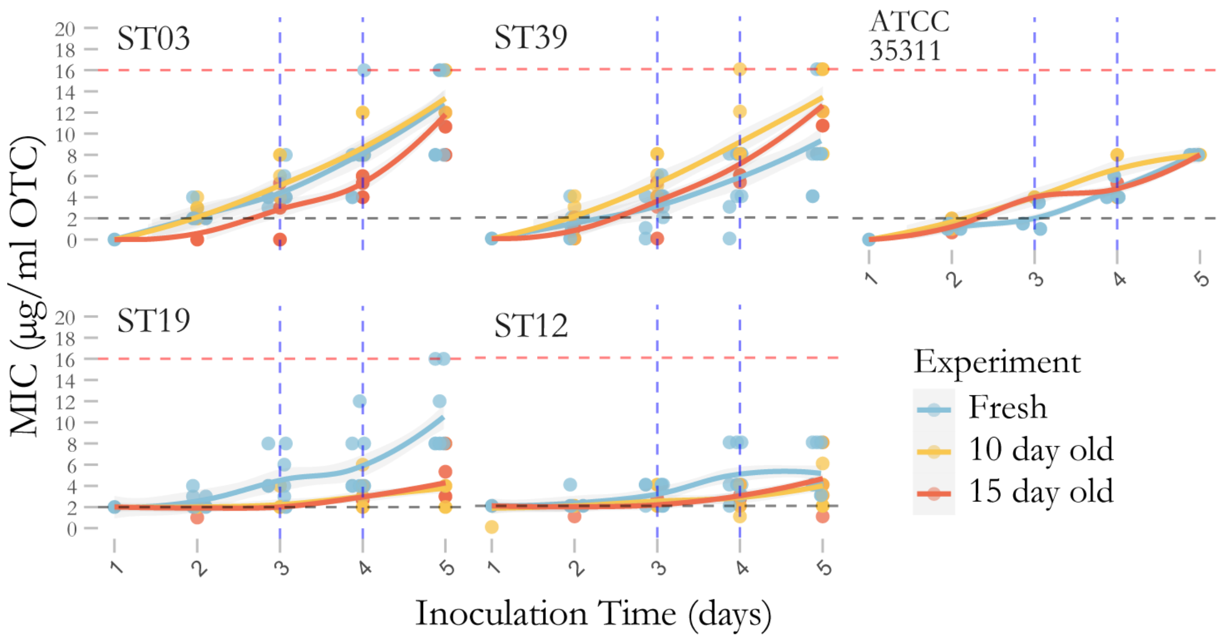

Figure S5. Minimum inhibitory concentration of *M. plutonius* to OTC in M110 agar prepared fresh (blue) compared with plates stored at 4°C for 10 (yellow) and 15 days (red) prior to inoculation. Vertical blue lines represent 72 and 96 hours when previous assays were stopped. Grey line at 2 represents when growth on antibiotic free control is first observed. Horizontal dashed red line at 16 represents the currently accepted MIC for OTC. Each dot represents one of three replicates.

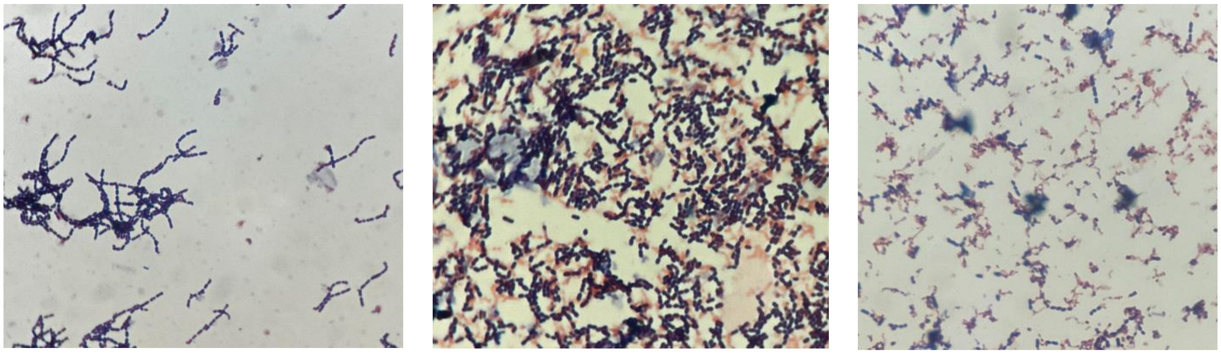

(a) **(b)** ©

Figure S6. Gram stains prepared during at 24 hours during growth analysis of ST12 shown in manuscript Figure 3a. a) Isolate 22O shows all cells intact staining gram positive at peak turbidity. b) Isolate 22N after mild decrease in turbidity showing significant gram-positive and gram-negative debris. c) Isolate 19P after a dramatic drop in turbidity shows mostly gram-negative debris.
